# Antioxidant and Antibacterial Screening and Hg(II) Sensing, Activities of Cu(II)pyridine‐2,6‐dicarboxylate Complexes

**DOI:** 10.1002/open.202400089

**Published:** 2024-07-25

**Authors:** Hameed Ur Rahman, Ezzat Khan, Mian Muhammad, Maaz Khan, Mashooq Ahmad Bhat, Gul Shahzada Khan, Nisar Ali

**Affiliations:** ^1^ Department of Chemistry University of Malakand 18800 Chakdara, Dir (Lower), Khyber Pakhtunkhwa Pakistan; ^2^ Department of Chemistry College of Science University of Bahrain Main Campus 32038 Sakhir Kingdom of Bahrain; ^3^ Department of Pharmaceutical Chemistry College of Pharmacy King Saud University 11451 Riyadh Saudi Arabia; ^4^ Key Laboratory for Palygorskite Science and Applied Technology of Jiangsu Province Faculty of Chemical Engineering National and Local Joint Engineering Research Centre for Deep Utilization Technology of Rock-Salt Resource Huaiyin Institute of Technology 223003 Huaian China

**Keywords:** copper(II) complexes, thermal properties, Hg(II) detection, antioxidant potential, antibacterial

## Abstract

In this study five different complexes of Cu(II) were synthesized for the purpose of environmentally notorious mercury sensing and preliminary biological screening. Pyridine‐2,6‐dicarboxylic acid (also known as dipicolinic acid, and abbreviated as H_2_DPA), 3‐phenyl pyrazole (3‐ppz), 4‐iodo‐1*H*‐pyrazole (4‐ipz), 4‐nitropyrazole (4‐npz), 4‐bromopyrazole (4‐bpz), and 4‐chloropyrazole (4‐cpz) were chosen as potential ligands. The synthesized complexes labelled as **1**–**5**, namely [Cu(DPA)(3‐ppz)], [Cu(DPA)(4‐ipz)], [Cu(DPA)(4‐npz)], [Cu(DPA)(4‐bpz)], [Cu(DPA)(4‐cpz)], were proposed based on spectroscopic data (FTIR, TGA, and UV‐visible spectroscopy). These complexes feature C=O functionalities that are not involved in coordination and may be used for further applications. The isolated complexes were utilized for detecting Hg(II) ions in water samples. Various concentrations of Hg(II) ions were prepared for detection purposes, and changes in absorption concerning complexes **1**–**5** were determined using UV‐Visible spectroscopy. It was found that complexes **3** and **4** exhibit efficient sensing abilities towards Hg(II) ions. The antibacterial activities of complexes **1**–**5** were assessed against *S. typhi* and *E. coli*. The complexes **1** and **3** displayed good antibacterial activities against *S. typhi* (13.67, and 13.56 mm, respectively) while complexes **1**, **2** and **4** were found to be efficient against *E. coli* (11.6, 12.66, 11.31 mm, respectively). The absorption maxima of 2,2‐diphenyl‐1‐picryhydrazyl (DPPH) at 517 nm, considerably shifted upon addition of complexes **1**–**5**. The results reveal that the complexes possess potential free radical scavenging abilities.

## Graphical abstract

Five complexes of Cu(II) are synthesized for mercury sensing and biological screening. Pyridine‐2,6‐dicarboxylic acid, 3‐phenylpyrazole (3‐ppz), 4‐iodo‐1*H*‐pyrazole (4‐ipz), 4‐nitropyrazole (4‐npz), 4‐bromopyrazole (4‐bpz), and 4‐chloropyrazole (4‐cpz) were chosen as potential ligands. The synthesized complexes were used for detecting Hg(II) ions in water samples. The antibacterial activities against *S. typhi*, *E. coli* and antioxidant potential of compounds are promising.

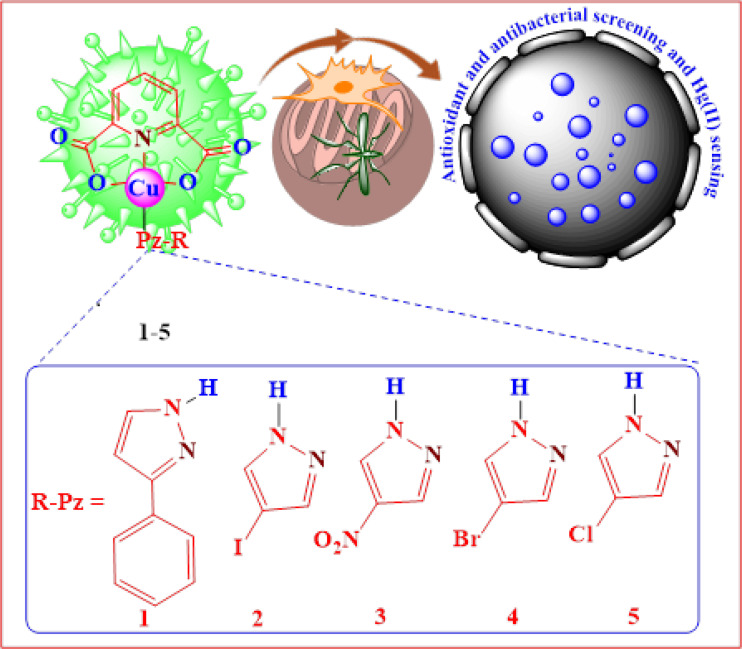

## Introduction

Copper complexes with various ligands are extensively reported in the literature.^[1]^ These complexes find diverse applications in biological systems and are of interest in both fundamental research and practical applications. Copper complexes have shown promising potential as anticancer agents,^[2]^ capable of targeting cancer cells through the inhibition of specific enzymes or the disruption of DNA synthesis and repair processes. Copper bis(thiosemicarbazone) complexes exhibit cytotoxic activity against various cancer cell lines.^[3]^ Additionally, some of these complexes exhibit potent antimicrobial properties against a wide range of microorganisms.^[4]^ They can disrupt microbial membranes, interfere with cellular processes, and generate reactive oxygen species that induce oxidative damage to microbial cells.^[5]^ Copper complexes have been explored as potential alternatives to traditional antibiotics, exhibit excellent photophysical properties and are being regarded as good candidates as bioimaging probes.^[6]^ The intense luminescence exhibited by copper complexes allows for sensitive detection and imaging of biological targets.^[7]^ These complexes are often used as fluorescent dyes for visualizing specific biomolecules or cellular structures.^[8]^ They inhibit enzymes by binding to their active sites and interfering with their catalytic functions.^[9]^ For example, copper complexes have recently been investigated against metalloenzymes to combat diseases such as Alzheimer's and Parkinson's.^[10]^ By modulating the activity of these enzymes, copper complexes can potentially provide therapeutic benefits.^[11]^ This type of complexes is known for their ability to undergo reversible electron transfer reactions, acting as electron transfer mediators in biological systems^[12]^ and participating in redox processes essential for various biological functions. In bioelectrochemistry and bioenergetics research copper complexes have been employed to understand electron transfer mechanisms and develop bioelectronic devices.^[13]^ The reactivity and structural features of copper‐containing metalloproteins, such as copper based enzymes like cytochrome c oxidase and copper‐zinc superoxide dismutase play very important role in biochemistry.^[14]^ By synthesizing copper complexes that mimic these metalloproteins, we can gain insights into the properties and roles of coper ions in biological processes. It's important to note that while Copper complexes have shown promising biological applications, their potential use in therapeutic or diagnostic contexts requires extensive research and development to ensure safety, efficacy, and specificity.^[15]^ The thermal properties of copper complexes can vary depending on the type of ligands, coordination geometry around the metal ion, and several other contributing actors.^[16]^ Some copper complexes demonstrate thermal stability and can retain their structural integrity at relatively high temperature, while others may decompose or undergo structural changes when heated. The thermal stability can be influenced by the nature of ligands and the coordination environment around the metal center.^[17]^

**Scheme 1:**
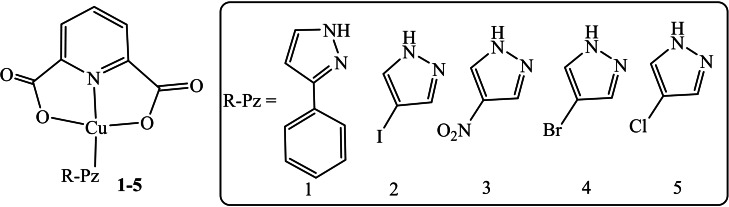
Structure of complexes **1**–**5**, obtained in this study.

Keeping in view the interesting properties of copper complexes,^[18]^ five copper complexes were synthesized in this study, with copper serving as the central metal ion (Scheme 1). These complexes were isolated in the solid state and characterized using FT‐IR, UV‐visible spectroscopy and their thermal behavior was assessed via TGA. All complexes underwent screening for preliminary biological activities, including antimicrobial and antioxidant potentials and their ability to detect Hg(II) ion was explored.

## Experimental Section

Chemicals used in this study, Cu(II) acetate, pyridine‐2,6‐dicarboxylic acid (dipicolinic acid abbreviated as H_2_DPA), 3‐phenylpyrazole, 4‐iodo‐1*H*‐pyrazole, 4‐nitropyrazole, 4‐bromopyrazole, 4‐chloro pyrazole, commercial grade methanol and ethanol, were purchased from Sigma Aldrich and TCI‐Japan and were used as received. The FT‐IR spectra of synthesized complexes were recorded on a (L1600300 TWO Lita) spectrophotometer in the range of 4000–400 cm^−1^. For thermogravimetric analyses (TGA‐50 SHIMADZU) thermal analyzer was used with temperature range 25–600 °C at a heating rate 30 °C/min under nitrogen atmosphere. The UV‐visible spectra were recorded by (SHIMEADZU UV‐4000) spectrophotometer.

Five different complexes using Cu(II) acetate as starting material were synthesized following the literature procedure.[^18^, ^19^] Separate solutions of an equimolar amount of Cu(II) acetate (0.050 g, 0.40 mmol) and DPA (0.0681 g, 0.40 mmol) were slowly mixed under vigorous stirring. To this mixture three‐fold excess (1.20 mmol) of respective amine base was slowly added, 3‐phenylpyrazole (0.176 g) **1**, 4‐iodo‐1*H*‐pyrazole (0.237 g) **2**, 4‐nitropyrazole (0.138 g) **3**, 4‐bromopyrazole (0.180 g) **4** and 4‐chloropyrazole (0.145 g) **5**. All solutions were prepared in 1 : 1 ethanol‐methanol solvent system (v/v). After mixing the reacting substances, the solution was heated to reflux for 8 h. The solutions were cooled to room temperature and where filtered. The filtrate was allowed to evaporate at room temperature, after 2–3 weeks, crystals appeared and were separated from the mother liquor.

Complex **1**: Yield**=**73 % (0.1292 g); m.p. (°C)=298–300; color: blue; UV data (nm)=248, 602 nm; FT‐IR (ATR) ν(cm^−1^)=3230, 3130, 1631, 1370, 1340, 592.

Complex **2**: Yield=71 % (0.1404 g); m.p. (°C)=302–305; color: blue; UV data (nm)=168, 276, 667; FT‐IR (ATR) ν(cm^−1^)=3360, 3098, 1648, 1427, 1336, 585.

Complex **3**: Yield=68 % (0.113 g); m.p. (°C)=315–318; color: blue; UV data (nm)=168, 276; FT‐IR (ATR) ν(cm^−1^)=3200, 3002, 1636, 1501, 1342, 590.

Complex **4**: Yield=70.7 % (0.126 g); m.p. (°C)=277–280; color: blue; UV data (nm)=161,276; FT‐IR (ATR) ν(cm^−1^)=3359, 3108, 1643, 1345, 585.

Complex **5**: Yield=72.6 % (0.125 g); m.p. (°C)=301–304; color: blue; UV data (nm)=267, 276, 690; FT‐IR (ATR) ν(cm^−1^)=3301, 3101, 1620, 1382, 595.

The FT‐IR absorption bands of different functional groups were observed for complexes **1**–**5**, and summarized in Table 2. From the data various stretching frequencies corresponding to the pyridine‐2,6‐dicorboxylate anion and pyrazole derivatives are present and confirmed by the appearance in the specified region in FT‐IR spectrums. The FT‐IR spectrums of complexes **1**–**5** have two distinct regions. In higher energy region the absorption bands from 3400–3100 cm^−1^ are due to N−H of pyrazole and aromatic C−H of pyridine ring. In lower energy region from 1600–450 cm^−1^ the peaks are assigned to *ν_ass_* (−C=O), *ν_sym_* (C−O), Cu−N and Cu−O bonds.

### Biological Screening of Complexes 1–5

Three gram‐negative bacterial strains were selected for *in‐vitro* antimicrobial potentials of complexes synthesized in this. The average numbers of microbes i. e., *Salmonella typhi* (ATCCO650), *Escherichia coli* (ATCC25922) and *Shigella* (AO30) per mL of the stock suspension were assessed by means of surface viable technique.^[20]^ About 10^8^–10^9^ colony forming per mL were considered and freshly prepared stock solution was used in each experiment. The experimental condition was optimized and maintained constantly so that suspension with very close viable counts would be obtained. The agar solution as growth medium was prepared and incubated under normal conditions, allowing the bacterial strains to proliferate. Once the incubation was complete, the agar solution was sterilized to eliminate any unwanted contaminants. After sterilization, the solution was left to solidify, forming a gel‐like substance on the plate surface. With the help of a sterile borer, holes were made wherein 100 μL of sample of each text complexes were applied in separate holes. The samples are left to diffuse into the agar for 24 h, giving enough time for any antibacterial substances to exert their effects and to grow. After the incubation period of 24 h, the zones of inhibition (ZI), which represent the area free from bacterial growth, were observed around the respective sample. The diameter of each ZI was measured in millimeters. Experiments were performed in triplicate and average values of ZI were tabulated.

The free radical scavenging capacities of complexes **1**–**5** was assessed against DPPH. A known amount, 0.380 g of DPPH was dissolved in 100 mL in methanol and was stored in dark for further tests. Methanol solution of test complexes was prepared with varying concentrations, 10, 20, 40, 80 and 160 ppm. To each sample's solution 1 mL of freshly prepared DPPH solution was added and the resultant DPPH/complexes mixtures was incubated under dark condition for 30 min. The total volume of DPPH/complex mixture was 2 mL. The absorption of DPPH/complex mixture was measured at 517 nm and the resultant decrease in absorbance was measured. The antioxidant activity of complexes **1** and **2**, percent inhibition of DPPH was calculated by the formula ***I %=(A***_***control***_ **−*A***_***sample***_ ***/A***_***control***_).^[21]^

### Hg(II) Detection Studies

The sensing capability of synthesized complexes **1**–**5** was determined against Hg(II) ion according to the reported procedures.^[22]^ The 100 μg/mL stock solution of Hg(II) salt was prepared by dissolving 0.014 g of HgCl_2_ in 10 mL water followed by dilution to 100 mL. For further dilution, the solution of 5.0 ppm was prepared by taking 5.0 mL of 100 μg/mL stock solution and was diluted up to 100 mL with distilled water. This 5.0 μg/mL stock solution was used for preparing working standard solutions. Similarly, 100 μg/mL stock solution of complexes **1**–**5** were prepared by dissolving 0.010 g of each complex in 10 mL of ethanol and then diluted to 100 mL using ethanol. In a series of 10 mL volumetric flasks 0.5 mL of 100 μg/mL were mixed with 0.1, 0.3, 0.7 and 1.0 mL of 5.0 μg/mL Hg(II) solution and diluted with distilled water. The spectra of Hg(II)/samples were recorded UV‐vis spectroscopy wherein quenching of enhancement was an evidence of the sensing ability of the respective complexes.

## Results and Discussion

**Figure 1:**
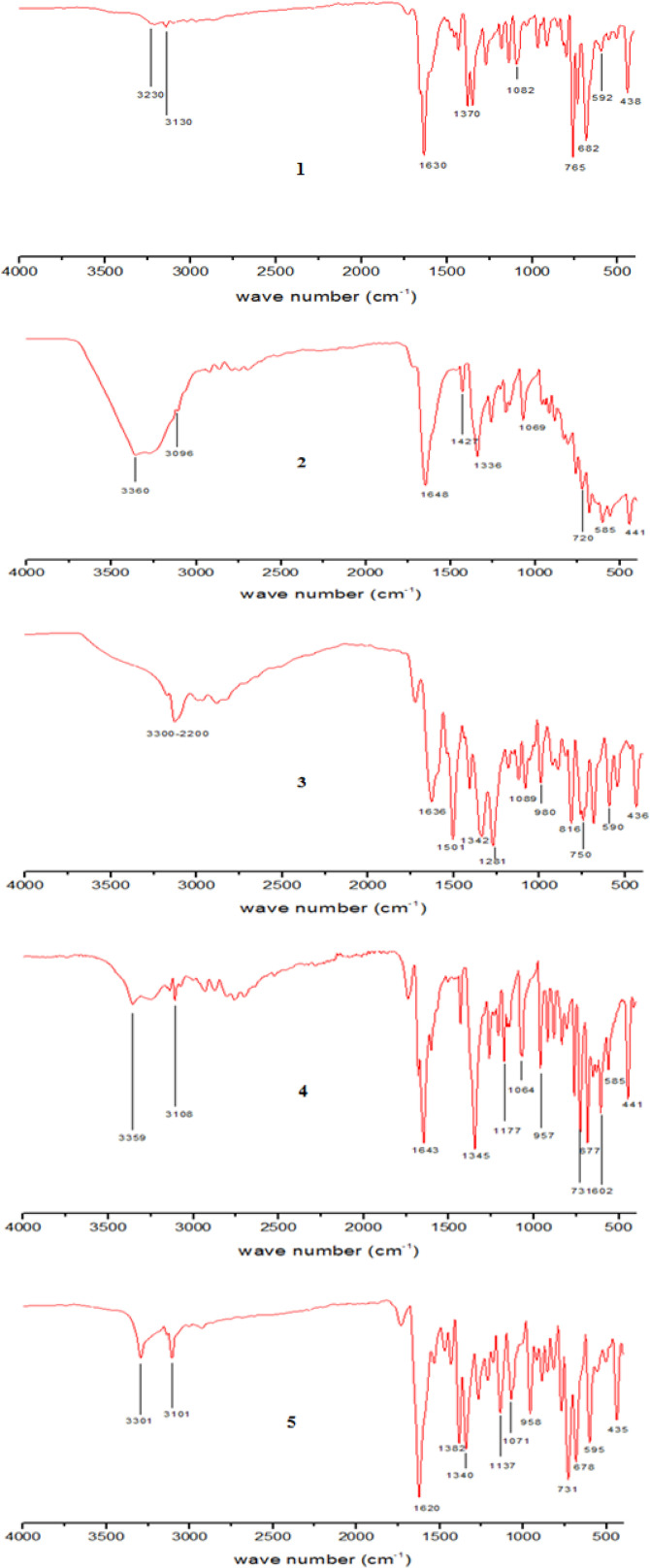
FT‐IR spectra of complexes **1**–**5**.

**Figure 2:**
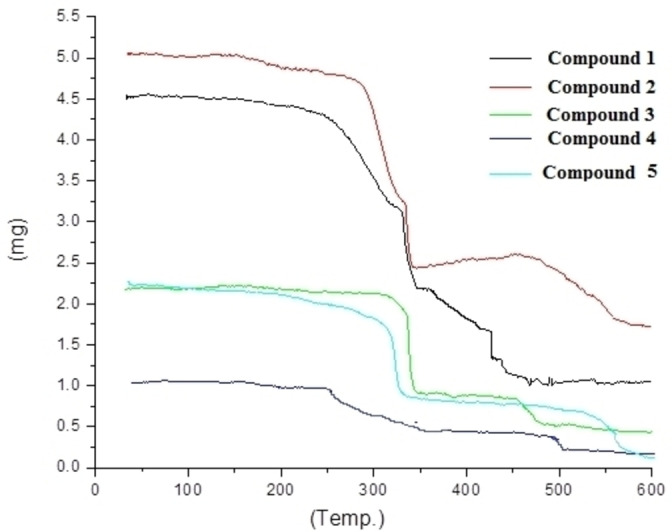
TGA curves of synthesized complexes **1**–**5**.

The experimental strategy was successful the synthesized coordination complexes were characterized by FT‐IR, UV‐vis spectroscopy (Figure 1) and TGA analysis (Figure 2). The electronic spectra of selected pyrazole derivatives (3‐phenylpyrazole, 4‐bromopyrazole, 4‐iodopyrazole, 4‐nitropyrazole and 4‐chloropyrazole) and H_2_DPA in it uncoordinated state were recorded in DMSO. The absorptions maxima of each complex as its DMSO solution at normal temperature were noted. The bands observed at 602 (ϵ 158.2 Lmol^−1^ cm^−1^), 667 nm (ϵ 149.2 Lmol^−l^ cm^−1^), 670 nm (ϵ 130.38 Lmol^−1^ cm^−1^), 732 nm (ϵ 150.73 Lmol^−1^ cm^−1^) and 690 nm (ϵ 131.70 Lmol^−1^ cm^−1^) for **1**–**5**, respectively can be assigned as d‐d transitions (Table 1). The complex **1** have distorted square pyramidal.^[23]^ The geometries of complexes **2** and **4** are distorted square planar according to the literature.^[24]^ If compared with literature data, complexes **3** and **5** possess distorted trigonal bipyramidal geometry around the central metal atom.[^24^, ^25^]

**Table 1:** UV‐Visible spectroscopic data of complexes and their starting precursors under identical conditions.

Complexes		π→π*nm	ε (Lmol^−1^ cm^−1^)	n→π*	d–d	ε (Lmol^−1^ cm^−1^)
H_2_DPA		271	4322	276	–	–
3‐ppz		247	4560	–	–	–
4‐ipz		225	4677	–	–	–
4‐npz		273	4153	–	–	–
4‐bpz		222	4208	–	–	–
4‐cpz		221	4385	–	–	–
**1**	[Cu(DPA)(3‐ppz)]	248	8596.2	–	602	158.2
**2**	[Cu(DPA)(4‐Ipz)]	268	6909.4	276	667	149.21
**3**	[Cu(DPA)(4‐npz)]	268	10194	276	670	130.38
**4**	[Cu(DPA)(4‐bpz)]	268	7356.2	276	732	150.73
**5**	[Cu(DPA)(4‐cpz)]	268	5429	–	690	131.70

### FT‐IR Measurements of Complexes 1–5

The FT‐IR spectrum of complexes **1**–**5** were measured in the range 200–4000 cm^−1^. In higher energy region the absorption bands at 3300–3380 cm^−1^ and 3104–3108 cm^−1^ are due to N−H of pyrazole and aromatic C−H, respectively for **1**–**5**. In low energy region a strong band at 1646 cm^−1^ are assigned to *ν_ass_* (C=O) while a strong band at 1345 cm^−1^ is due to *ν_sym_* (C−O) stretching of carbonyl bonds. Difference between symmetric and asymmetric C=O frequency (Δ *ν*) associated with DPA was found in the 245–277 cm^−1^ indicating that carboxyl groups are acting as mono‐dentate ligands (Table 2). Two bands at 593 and 438 cm^−1^ are observed within the expected region and can be assigned to Cu−N and Cu−O, respectively.^[26]^

**Table 2:** Stretching frequencies of various functionalities in the FT‐IR spectra of complexes **1**–**5**.

Compounds		N−H	Ar−C−H	Vas(C=O)	C=N	Vs(C−O)	Cu−N	Cu−O
H_2_DPA		–	3077	1701	1560	1456	00	00
**1**	[Cu(DPA)(3‐ppz)]	3230	3130	1630	1420	1348	592	438
**2**	[Cu(DPA)(4‐Ipz)]	3360	3098	1648	1427	1336	585	441
**3**	[Cu(DPA)(4‐npz)]	3370	3120	1636	1501	1342	590	436
**4**	[Cu(DPA)(4‐bpz)]	3359	3108	1643	1421	1345	585	441
**5**	[Cu(DPA)(4‐cpz)]	3301	3101	1620	1382	1340	595	435

### Thermal Properties of Synthesized Copper(II) Complexes 1–5

Thermal degradation of copper complexes varies widely depending on the coordinated ligands and structural features of the complex. TGA is a commonly used technique to analyze the thermal properties of compounds, including copper complexes.^[27]^ TGA provides information about the thermal stability, decomposition behavior, and the presence of readily volatile components in the complex.^[28]^ The thermal decomposition of copper complexes takes place through various pathways depending on ligands and coordination geometry. Decomposition involves processes namely, ligand dissociation, oxidation or reduction of the copper center, release of ligand fragments, or formation of new coordination species.^[29]^ Thermal decomposition pathways provide insights into the stability and reactivity of metal complexes. Such properties can be influenced by ligand substitution, counterions, solvent effects, and the presence of other molecules or ions. Therefore, a thorough investigation of the specific complex of interest is necessary to fully understand its thermal behavior.^[30]^

The TGA curves of complexes **1**, **4** and **5** give two stages of weight loss. The first weight loss occurs from 110–340 °C, which is 37.6 % (calculated value 38.5 %) for **1**, 38.3 % (calculated 38.74 %) for **4** and 30.8 % (calculated 30 %) for complex **5** corresponding to loss of 3‐phenypyrazole, 4‐bromo‐pyrazloe and 4‐chloro‐pyrazole, respectively. The second weight loss occurs from 348–495 °C, which is because of dissociation of DPA. The weight loss is 43.32 % (theoretical 44.23 %), for **1**, 43.9 % (theoretical 43.52 %) for **4** and 49.5 % (theoretical 50 %) for complex **5**, respectively. The residue weight indicated the copper(II) ion which gradually decomposes above 500 °C. The TGA curves of complexes **2** and **3** exhibit two stages of weight loss, similar to those observed for the previously mentioned complexes. In the first stage 39 % weight (theoretical 39.1 %) for **2** and 50 % weight (calculated 49.45 %) for **3** which correspond to DPA moiety. In the second stage 44 % (expected 43.79 %) weight loss was found which is because of 4‐Iodopyrazole for complex **2** and 32 % weight loss (expected 33.12 %) occur which corresponds to 4‐Nitropyrazole in complex **3** in range of 355–490 °C. The remaining weight loss for complexes **1**–**5** occurs above 500 °C.

### Antibacterial Study of Synthesized Copper(II) Complexes 1–5

**Figure 3:**
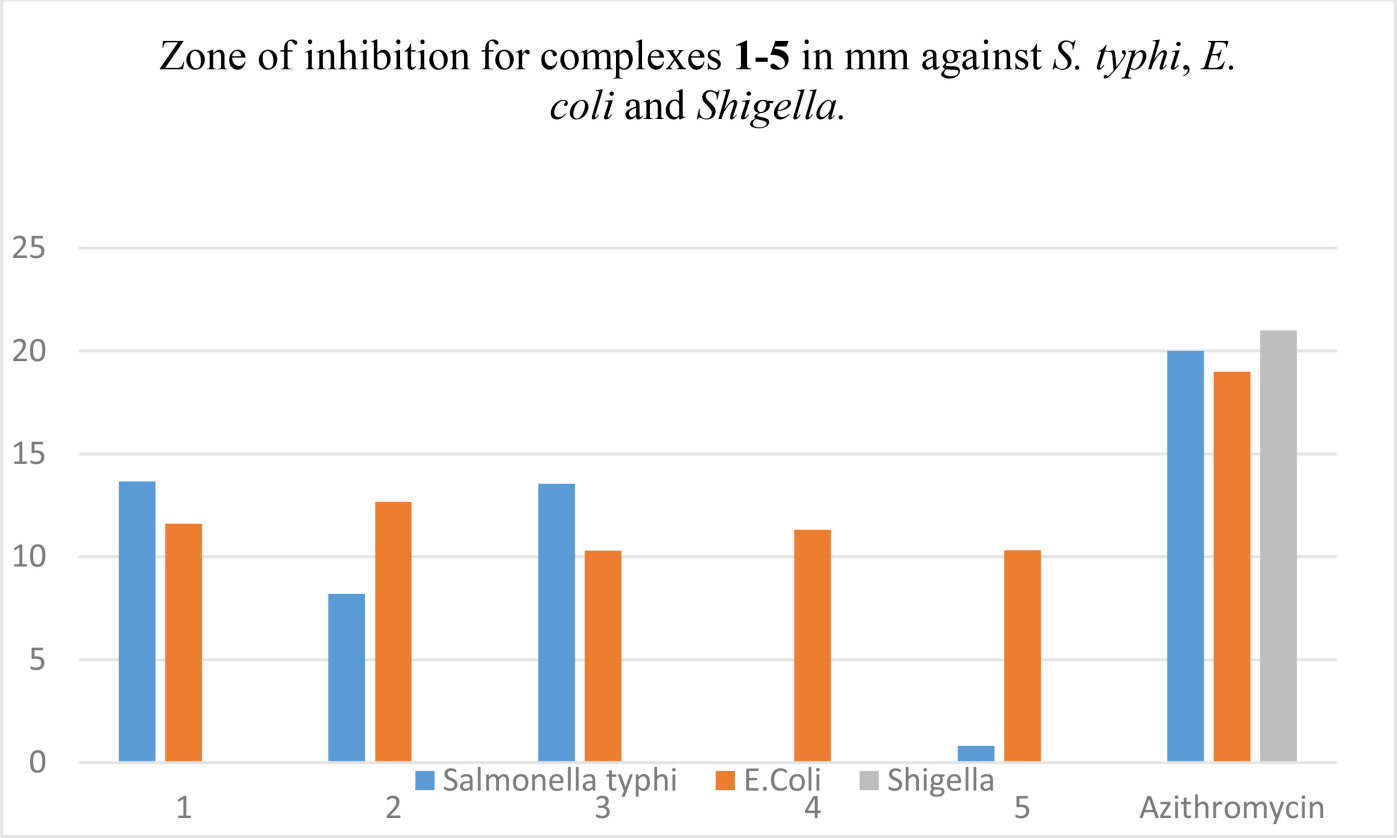
Bar graph for comparison of antibacterial efficiency of complexes **1**–**5**.

Complexes **1** and **3** exhibited good activity against *S. typhi* with the measured zone of inhibition 13.67 and 13.56 mm, respectively. complexes **2** and **5** were relatively less active against the same strain of bacteria *ZI* 8.2 and 0.81 mm, respectively while no activity was observed in case of complex **4**. Similarly complexes **1**, **2** and **4** showed relatively good activity against *E. coli* with ZI 11.6, 12.66 and 11.31 mm, respectively while **3** and **5** were least active (ZI 10.31 and 10.32 mm, respectively). All complexes **1**–**5** are inactive against *shigella* as shown in bar graph Figure 3. Azithromycin was used as a standard, the measured zone of inhibition 20, 19, and 21 mm for *S. typhi*, *E. coli* and *Shigella* respectively.

### Antioxidant Properties of Synthesized Copper(II) Complexes 1–4

The quantitative antioxidant activity was determined by reduction in the absorption peak intensity of pure DPPH at 517 nm. The complexes **1**, **2** and **5** give naked eye and high free radical scavenging capacities, a sudden change occurs in the color of DPPH solution from purple to yellow. The effect was almost the same for higher and lower concentration of the complexes which reveals excellent scavenging efficiency IC_50_ 4.74, 5.32 and 5.31 ppm respectively. For complexes **3**–**4** color change cannot be seen but gradual decrease was observed for absorption bands at 517 nm with increase the concentration of test complexes **3**–**4**. The obtained results indicate that complexes **3** and **4** show greater activity even at its minimum concentration i. e. 10 ppm. This decrease can be considered a governing factor for determination of antioxidant potency. The percent inhibition and IC_50_ values of complexes are tabulated in Table 3.

**Table 3:** Percent inhibition and IC_50_ valve of complexes **1**–**4**.

S. NO	complexes	IC_50_ (ppm)	Percent inhibition
**1**	[Cu(DPA)(3‐ppz)]	4.74	88.3
**2**	[Cu(DPA)(4‐Ipz)]	5.32	87.7
**3**	[Cu(DPA)(4‐npz)]	9.49	75.4
**4**	[Cu(DPA)(4‐bpz)]	9.12	78.3
**5**	[Cu(DPA)(4‐cpz)]	5.31	86.0
Standard	Ascorbic acid	30	97.98

### Detection of Mercury Hg(II)

**Figure 4:**
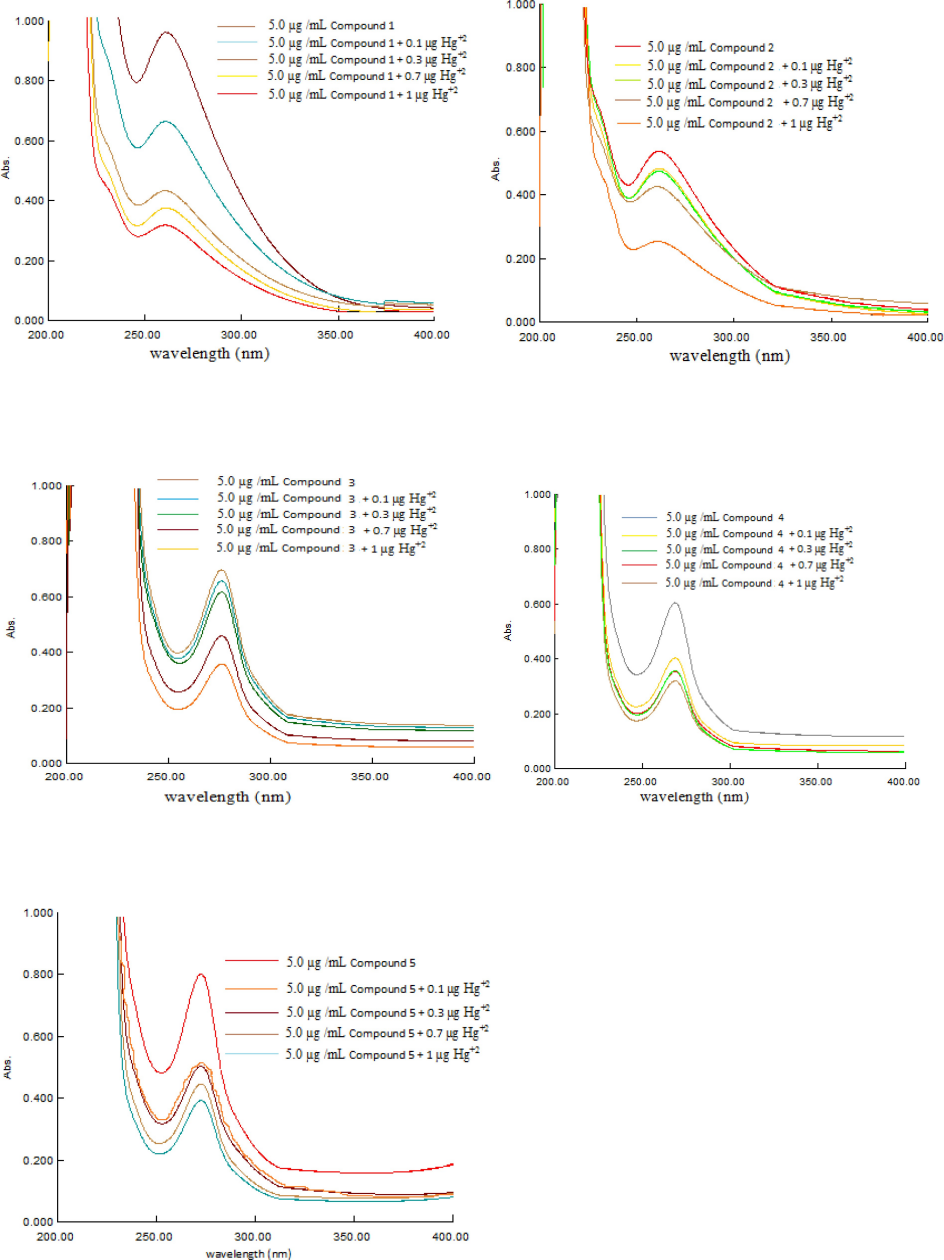
UV‐visible absorption spectra of complexes **1**–**5** (5 μg mL^−1^) as a function of concentration of Hg(II) (0.1, 0.3, 0.5, 0.7 and 1 μg mL^−1^).

Metal complexes have the potential to be used for several applications including their potential and sensory molecules.^[31]^ Following the same concept, all the Cu(II) complexes (**1**–**5**) were used as chemosensor for indirect determination of Hg(II). It was observed that absorbance of the complexes shows indirect relation to the concentration of Hg(II). Variable concentration of Hg(II) (0.1 to 1 μg mL^−1^) was added to solution of each complex (5 μg mL^−1^) in 10 mL volumetric flasks and diluted up to the mark with ethanol. The resulting mixtures were kept for 30 min at room temperature to be equilibrated and then absorbance was recorded at the respective λ_max_ of the complexes **1**–**5**. The absorbance in all cases was found to decrease linearly with increasing concentration of Hg(II) as shown in Figure 4. These changes correspond to interaction of oxygen and nitrogen atoms present in compounds **1**–**5**, with Hg(II) ion. Relative absorbance was plotted against the concentration of Hg(II) and slope of curve was used for subsequent calculation of analytical parameters including limit of detection and limit of quantification. To proceed further replicate analysis of the lowest concentration of Hg(II) which can be accurately quantified i.e (0.1 μg mL^−1^) was carried out and sample standard deviation was calculated in each case. The limit of detection and limit of quantification of the proposed method using the mentioned complexes were calculated with the help of the following formula (Eq. 1 and 2.^[32]^

(1) $$\mathrm{LOD}=\frac{3.3\sigma }{slope\;of\;calibration\;curve}\;$$

(2) $$\mathrm{LOQ}=\frac{10\;\sigma }{slope\;of\;calibration\;curve}\;$$

Where σ in both equations, is the standard deviation of the proposed sensing method for determination of Hg(II). The SD, LOD and LOQ values for determination of Hg(II) using the respective complexes **1**–**5** are given in Table 4.

**Table 4:** LOD and LOQ of complexes **1**–**5** in mercury ion sensing. LOD was measured in unit of μg/mL.

Sample	Complex	Standard deviation	LOD	LOQ
[Cu(DPA)(3‐ppz)]	**1**	0.021	0.044	0.112
[Cu(DPA)(4‐ipz)]	**2**	0.022	0.034	0.103
[Cu(DPA)(4‐npz)]	**3**	0.021	0.070	0.203
[Cu(DPA)(4‐bpz)]	**4**	0.010	0.055	0.165
[Cu(DPA)(4‐cpz)]	**5**	0.001	0.040	0.123

**Figure 5:**
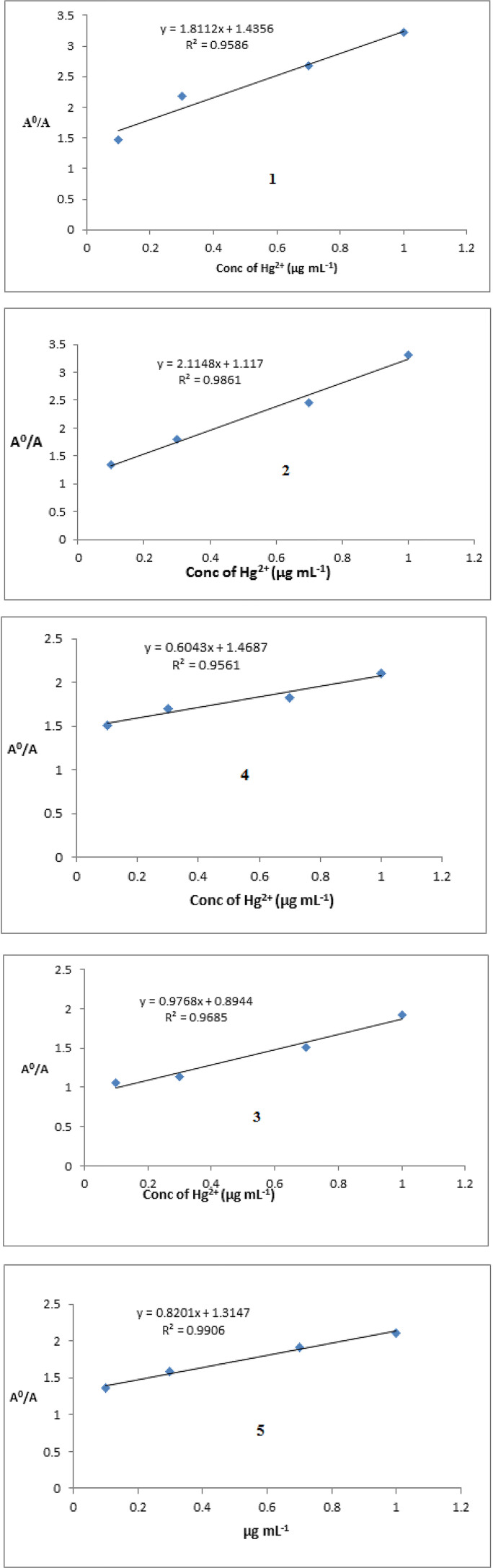
Plots between A_o_/A vs concentration of Hg(II) ions for complexes **1**–**5** where A_o_=Absorption of pure complex, and ACComplex+Hg(II) ions.

The graphs shown in Figure 5, A^o^/A Vs concentration of Hg(II) ion is plotted, the straight equations for each complex **1**–**5** is obtained. The LOD and LOQ were calculated using the formulas given above. The respective values for LOD and LOQ are given in Table 4. The A^o^ represent the absorbance of complex alone and ‘A’ represents the absorbance of complex and Hg(II) ion mixture.

## Conclusions

Five distinct Cu(II) complexes with the specific aim of mercury sensing were synthesized, using starting materials, Cu(II) acetate and various pyrazole derivatives, including H_2_DPA, 3‐phenylpyrazole (3‐ppz), 4‐Iodo‐1*H*‐pyrazole (4‐ipz), 4‐Nitropyrazole (4‐npz), 4‐bromopyrazole (4‐bpz), and 4‐Chloropyrazole (4‐cpz). The resulting complexes, with general representation [Cu(DPA)(3‐ppz)], [Cu(DPA)(4‐ipz)], [Cu(DPA)(4‐npz)], [Cu(DPA)(4‐bpz)], and [Cu(DPA)(4‐CPz)] were employed for detecting Hg(II) ions. To detect Hg(II) ions, the solution of each Cu(II) complex were mixed with various concentrations of Hg(II) and changes in absorption relative to the sensory complex was determined by UV‐visible spectroscopy. Interestingly, complexes **3** and **4** exhibited highly efficient sensing capabilities for Hg(II) ions, making them particularly promising candidates for further investigation. Moreover, we assessed the antibacterial activities of complexes **1**–**5** against *S. Typhi* and *E. Coli* bacteria, aiming to explore their potential in combating bacterial infections and were found of moderate efficiency.

## Funding

Research supporting project number (RSPD2024R740), King Saud University, Riyadh, Saudi Arabia.

## Author Contributions

Conceptualization, H. R. and E. K.; methodology, H. R.; software, M. M. and M. K.; validation, E. K., M. M. and A. N.; investigation, H. R.; resources, E. K., M. M., G. S. K. and N. A.; writing – original draft preparation, H. R., M. M., A. N. and E. K.; writing – review and editing, E. K. and A. N.; visualization, H. R., M. K. and E. K.; supervision, E. K.; project administration and funding, M. A. B. All authors have read and agreed to the published version of the manuscript.

## Conflict of Interests

The authors declare no conflict of interest.

1

## Data Availability

Data sharing is not applicable to this article as no new data were created or analyzed in this study.
